# Identifying set-wise differential co-expression in gene expression microarray data

**DOI:** 10.1186/1471-2105-10-109

**Published:** 2009-04-16

**Authors:** Sung Bum Cho, Jihun Kim, Ju Han Kim

**Affiliations:** 1 Seoul National University Biomedical Informatics (SNUBI), Seoul National University College of Medicine, Seoul 110-799, Korea; 2 Interdisciplinary Program in Bioinformatics, Seoul National University, Seoul 151-747, Korea

## Abstract

**Background:**

Previous differential coexpression analyses focused on identification of differentially coexpressed gene pairs, revealing many insightful biological hypotheses. However, this method could not detect coexpression relationships between pairs of gene sets. Considering the success of many set-wise analysis methods for microarray data, a coexpression analysis based on gene sets may elucidate underlying biological processes provoked by the conditional changes. Here, we propose a differentially coexpressed gene sets (dCoxS) algorithm that identifies the differentially coexpressed gene set pairs between conditions.

**Results:**

dCoxS is a two-step analysis method. In each condition, dCoxS measures the interaction score (IS), which represents the expression similarity between two gene sets using Renyi relative entropy. When estimating the relative entropy, multivariate kernel density estimation was used to model gene-gene correlation structure. Statistical tests for the conditional difference between the ISs determined the significance of differential coexpression of the gene set pair. Simulation studies supported that the IS is a representative measure of similarity between gene expression matrices. Single gene coexpression analysis of two publicly available microarray datasets detected no significant results. However, the dCoxS analysis of the datasets revealed differentially coexpressed gene set pairs related to the biological conditions of the datasets.

**Conclusion:**

dCoxS identified differentially coexpressed gene set pairs not found by single gene analysis. The results indicate that set-wise differential coexpression analysis is useful for understanding biological processes induced by conditional changes.

## Background

Microarray data analysis is important for evaluating global gene expression profiles and has been widely applied to functional genomics. It enables identification of disease marker genes [1-3] and gene expression regulatory networks [4-6]. It can also be used to evaluate evolutionary conservation of gene coexpression [7].

Among the microarray data analysis methods, coexpression analysis has provided information about genetic regulatory relationships [8-10]. Cluster analysis can also be considered coexpression analysis, determining correlated groups of genes that are tightly coregulated [11].

In contrast to coexpression analysis that determines the degree of coexpression of a gene pair or gene set under a certain condition, differential coexpression analysis determines the difference in coexpression under different conditions, which may relate to key biological processes provoked by changes in environmental conditions [12-16].

Differential coexpression analysis can be divided into two types. The first identifies a gene pair that has significant coexpression differences between conditions. For example, Lai *et al. *identified differentially coexpressed gene pairs using expected conditional *F*-statistic (*ECF*), a modified *F *statistic [13]. Choi *et al. *detected gene pairs with significant differential coexpression between normal and cancer samples through a meta-analytic approach [14]. The second type of differential coexpression determines whether a gene cluster (or set) shows significant conditional differences in the degree of coexpression between genes in that cluster. To measure the degree of coexpression under each condition, Kostka and Spang used an additive model-based scoring system [15], and Watson used t-statistics [16].

No method, however, determines whether a pair of gene sets shows significant difference in expression profiles under different conditions. In single gene pair analysis, detection of the differentially coexpressed pairs led to varying hypotheses associated with biological or experimental conditions. Likewise, differentially coexpressed gene set pairs may be related to biological processes induced by certain conditions. For example, if p53 signaling and tumor necrosis factor (TNF) signaling pathways show differential coexpression in diseased conditions, the pathways are likely to be connected to the pathophysiology of the disease. Moreover, gene set-wise approaches are advantageous for microarray data analysis in terms of being better able to detect subtle changes and create biologically interpretable results than single gene-wise analysis [17]. Thus, to determine whether a gene set pair is differentially coexpressed under different conditions, we developed the dCoxS (differential coexpression of gene sets) algorithm, which has the benefits of both differential coexpression analysis and gene set-wise analysis.

**Figure 1:**
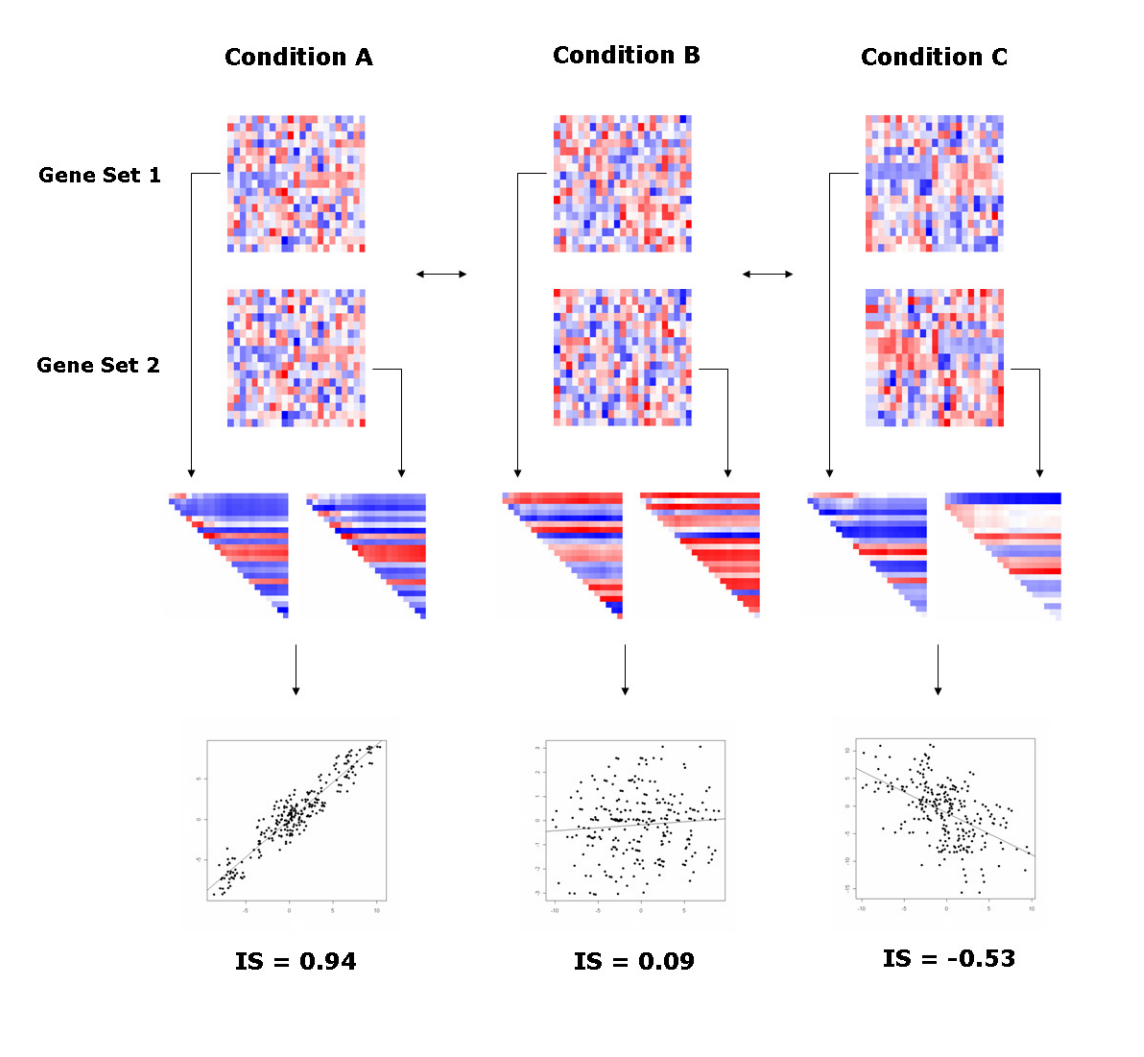
**Overview of the dCoxS algorithm**. Gene expression profiles of two gene sets are on the upper panel. In condition A, expression profiles of gene sets 1 and 2 are very similar. The similarity is reduced in condition B, and co-varies in a reverse way in condition C. The dCoxS quantifies the similarities and tests the significance of the change in the similarities across conditions. First, the sample-wise Renyi relative entropy matrix is obtained for each gene set. Then, the correlation coefficient of the upper-diagonal elements of the matrices, which represents the IS, is calculated for each condition. Diagonal heat maps in the middle represent the upper-diagonal elements of the sample-wise Renyi relative entropy matrices. The heat maps are transformed to the scatter plots in the lower part, and the fitted lines of the plots represent the ISs.

Here we define the differential coexpression of a gene set pair as a significant difference in the expression similarity of two gene sets under different conditions. The two gene set expression matrices consist of same samples and different genes. We used biological pathways as predefined gene sets and analyzed the differential coexpression of the biological pathway pairs between conditions. The dCoxS algorithm identified the differentially coexpressed pathway pairs through the following steps (See Figure 1). The expression similarity of two pathways was measured with an interaction score (IS) using the Renyi relative entropy. The Renyi relative entropy is equal to the distance between two samples. Because the two gene sets have the same sample sets, we obtained the same number of sample-wise distances from the two expression matrices. After the entropies of the two pathway expression matrices were calculated, the IS was determined by a correlation coefficient between the entropies of the two pathways. As the IS uses sample-wise distances, it can be calculated whether the two pathways contain the same genes or not. Finally, dCoxS analyzed the change in the ISs between conditions. The validity of dCoxS was evaluated with simulation datasets and two public microarray datasets.

## Results

### Simulation study

We used simulation data to evaluate whether the IS reflected the similarity between two expression matrices. Pathway expression matrices (*n *= 700 = 350 × 2) from two real datasets were used. For each gene set expression matrix, we generated six dissimilar expression matrices by adding random values generated from a normal distribution with different standard deviations (SDs; see methods). As the SD increased, the similarity between the original and the simulation data decreased because larger random values were added to the original matrices with the increased SDs. The Mantel statistics computed using five different metrics (Bray, Canberra, Euclidean, Gower and Manhattan) indicated that the average similarity between the original and the simulation data decreased with the increased SDs (Table 1). Like Mantel statistics with the five metrics, the mean IS decreased as the SD of the normal distribution increased. The differences of the mean ISs between different SD groups were statistically significant (p value < 0.001).

**Table 1:** Evaluation of distance measures by simulation study.

	*SD*					
	
Distance Metric	0.05	0.1	0.2	0.3	0.4	0.5
IS	0.9982	0.8633	0.6133	0.4232	0.3000	0.2367
Gower	0.9989	0.9177	0.7715	0.6178	0.5016	0.4128
Canberra	0.9994	0.9477	0.8314	0.6846	0.5567	0.4571
Bray	0.9995	0.9571	0.8575	0.7248	0.6056	0.5085
Manhattan	0.9995	0.9574	0.8587	0.7276	0.6102	0.5137
Euclidean	0.9998	0.9748	0.9031	0.7950	0.6819	0.5851

SD: standard deviation, IS: interaction score

We used the same simulation data to compare the IS and the Mantel statistics with the five different metrics. Table 1 shows that the mean IS had the lowest similarity score at a given SD. The differences between the IS and the other statistics were statistically significant at all SDs (p value < 0.001).

### Lung cancer data analysis results

We tested 61,075 pairs from the 350 pathways to find differentially coexpressed pathway pairs in the real data analysis. The IS of a pathway pair was computed for each condition, and the statistical significance of the difference in the ISs was tested.

In the lung cancer data analysis, we used a strict threshold (p value = 2.2E-16) to determine the significance of differential coexpression. Since 53% of p values from parametric tests were lower than the Bonferroni adjusted p value 8.187E-7 (*α *= 0.05, *n *= 61,075 gene set pairs), we chose the strict threshold to focus on more significant results. The threshold was one percentile of the p values obtained from dCoxS analysis of all pathway pairs. In the analysis, three p values were obtained from testing the ISs of the normal and the diseased conditions and testing the difference of the ISs between the samples. Even if the conditional difference of the ISs was significant, one of the IS p values from two conditions could be nonsignificant. Thus, we selected significant pairs only when all three p values were lower than the threshold.

Sixty-five (0.11%) of the 61,075 pathway pairs were significant within the criteria (see Table S1 in additional file 1). Thirty-eight of the 65 pairs did not have shared members. For the pairs with shared genes, we applied both assigning and nonassigning methods (see methods). Because we found no significant pairs using the nonassigning method, we chose the better assignment with a bigger difference of Fisher's Z-transformed IS (dZIS). The assigning method returned 27 significant pairs. All pairs were also statistically significant in the permutation test (p value < 8.0E-7). Table 2 shows the top 10 pathway pairs sorted by the dZIS value. The *Cytokine Network *and *TNF/Stress-related *pathway pair yielded the largest dZIS. The second largest dZIS was that of the *Estrogen-responsive protein Efp-*related and *Propanoate metabolism *pathway pair. Many important carcinogenesis-associated pathways such as cell cycle, apoptosis and telomerase pathways were found (see Table S1 in additional file 1).

**Table 2:** Top 10 pathway pairs showing significant differences in Z-transformed interaction scores in the lung cancer dataset.

Pathway pair	# of OGs	IS		dZIS
			
		NL	SCC	
Cytokine Network (37) TNF/Stress-Related Signaling (54)^§^	7	0.97	0.56	13.8

Estrogen-responsive protein Efp controls cell cycle and breast tumor growth (24) Propanoate_metabolism (26)	0	0.97	0.55	13.6

Activation of Src by Protein-tyrosine phosphatase alpha (22) Nuclear_Receptors (51)	0	0.96	0.59	12.2

Double-Stranded RNA-Induced Gene Expression (15) Neuroregulin receptor degradation protein-1 Controls ErbB3 receptor recycling (13)	0	0.96	0.56	11.8

Acute Myocardial Infarction (23) Angiotensin-converting enzyme 2 regulates heart function (18)^§^	11	0.96	0.60	11.5

ALK in cardiac myocytes (52) Inositol_phosphate_metabolism (146)	0	0.96	0.61	11.4

p38 MAPK Signaling Pathway (69) Apoptosis (73)^§^	10	0.95	0.54	11.3

fMLP-induced chemokine gene expression in HMC-1 cells (62)^§^ PTEN-dependent cell cycle arrest and apoptosis (25)	2	0.95	0.56	11.2

BRCA1-dependent Ub-ligase activity (18) Aminosugars_metabolism (13)	0	0.96	0.61	11.2

Endocytotic role of NDK, Phosphins and Dynamin (21) Pyruvate_metabolism (40)	0	0.96	0.65	11.0

# of OGs: number of overlapping genes in two gene sets, NL: normal lung, SCC: squamous cell carcinoma, dZIS: difference of Z-transformed IS, (): number of genes in a gene set, Nonparametric p value < 8.0E-7, ^§^: Shared genes are assigned to this pathway.

For gene pair-wise differential coexpression tests, the correlation coefficients of each gene pair were obtained, and the conditional difference of the correlation coefficients was tested using Bonferroni's multiple testing correction. In contrast to the set-wise analysis results, there were no significant results in the single gene differential coexpression analysis.

**Figure 2:**
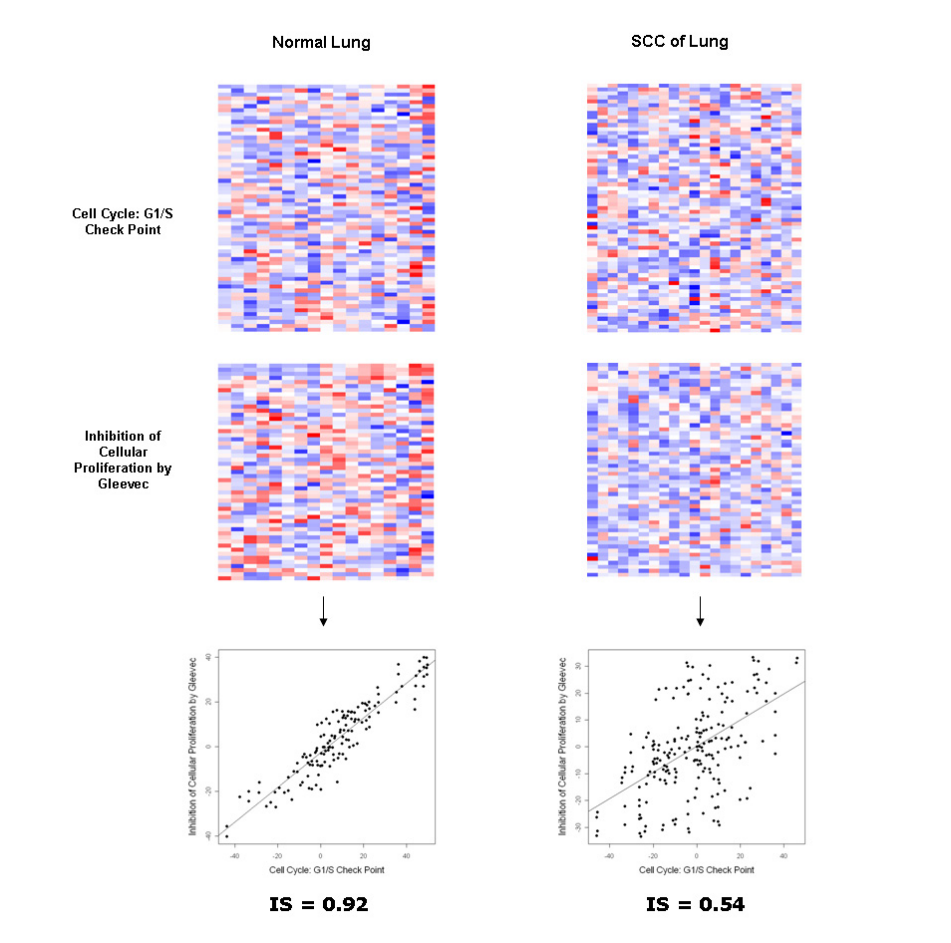
**The result of analysis of the *cell cycle: G1/S check points *and the *inhibition of cellular proliferation by Gleevec *pathway pair in the lung cancer dataset**. The upper and middle panels show gene expression profiles of the *cell cycle:G1/S check points *pathway and the *inhibition of cellular proliferation by Gleevec *pathway, respectively. The similarity between the pathways and the conditional change in the similarity are represented by the IS plots in the lower panel. Although the expression patterns of the raw pathway expression matrices appear to be more similar in normal lung samples, it is hard to quantify the change of the similarity in heat maps, whereas the similarity between the pathways and its conditional change is easily identified in the IS plots.

Figure 2 shows the expression profiles and the ISs of the *Cell Cycle: G1/S Check Point *and *Inhibition of Cellular Proliferation by Gleevec *pathways. The expression profiles of the pathways seem less similar in lung cancer than in normal lung samples by visual inspection. This difference is more evident in the IS scatter plots (Figure 2, lower panel).

The dynamic relationship between the differentially coexpressed gene sets can be expanded to construct a network of closely collaborating gene sets. Table 3 summarizes the five major pathways of the network, showing significant differential coexpression with several other pathways (see Table S2 in additional file 1 for the comprehensive list). The *Thrombin signaling and protease-activated receptors *pathway showed differential coexpression with five other pathways, which was the highest number of interacting pathways.

**Table 3:** Major pathways showing significant differential coexpression with other pathways in the lung cancer dataset.

Pathway	K	Sum(ZIS)
Thrombin signaling and protease-activated receptors (30)	5	43.9
Cell Cycle: G1/S Check Point (42)	4	39.0
Activation of Src by Protein-tyrosine phosphatase alpha (13)	3	31.6
TNF/Stress-Related Signaling (29)	3	31.5
Pyruvate_metabolism (18)	3	30.3

*K*: number of pathways showing differential coexpression, Sum(ZIS): total sum of Z-transformed IS, (): number of genes in a gene set.

### Duchenne's muscular dystrophy data analysis results

In the Duchenne's muscular dystrophy (DMD) data analysis, we used the tenth percentile of the p values (= 1.18E-8) obtained from the parametric test as a cutoff threshold because only three pairs of gene sets were significant within the one percentile threshold. Although we increased the p-value threshold, it was still lower than the Bonferroni adjusted p values (= 8.187E-7). When the threshold was applied as in the lung cancer data analysis, 30 pathway pairs were significant (see Table S3 in additional file 1). The results of the permutation test for the 30 pathway pairs were all significant (p value < 8.0E-7). Twenty-five of the 30 pairs did not have shared members. Of those that did have shared members, we found no significant pair using the nonassigning method; the assigning method returned five significant pairs with a one-way assignment (see Table S3 in additional file 1). As with the lung cancer data, single gene differential coexpression analysis detected no significant results.

**Figure 3:**
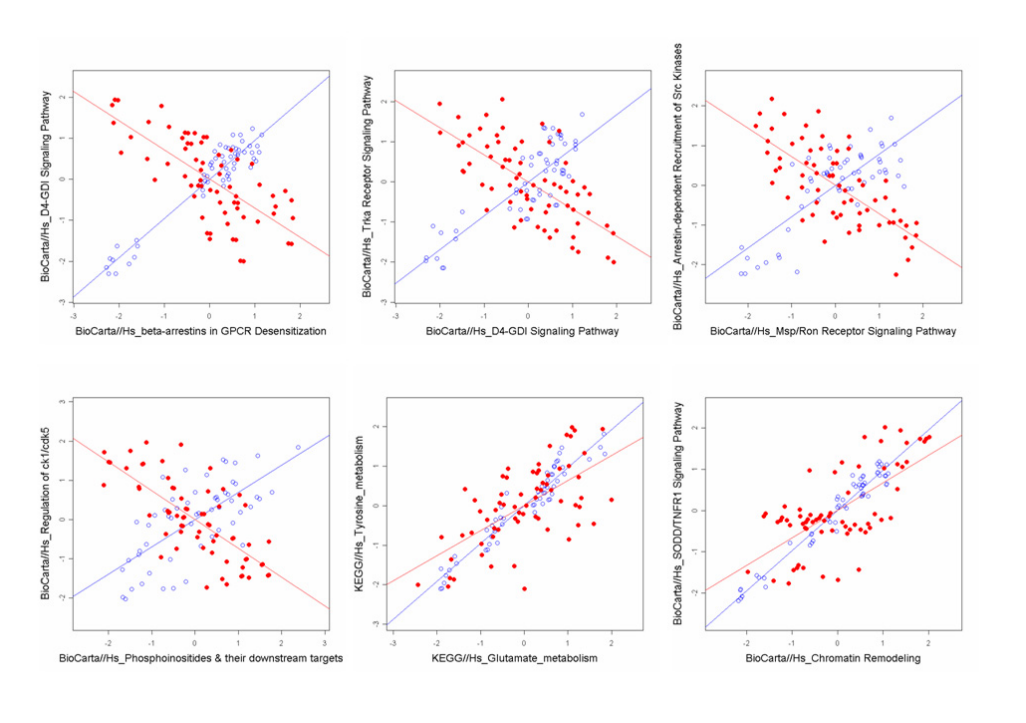
**Differentially coexpressed pathway pairs in the DMD dataset**. Blue and red indicate the normal and DMD samples, respectively.

Table 4 shows the pathway pairs that have the top 10 dZISs. The *Beta-arrestins in GPCR Desensitization *and *D4-GDI Signaling *pathway pair had the highest dZIS value. The *D4-GDI Signaling *and *Role of arrestins in the activation and targeting of MAP kinases *pathway pair had the second highest dZIS. Figure 3 shows the scatter plots of relative entropies and the ISs of the six selected pathway pairs, which may be related to the pathophysiology of DMD.

**Table 4:** Top 10 pathway pairs showing significant difference of Z-transformed interaction scores in the DMD data.

Pathway Pair	# of OGs	IS		dZIS
			
		NM	DMD	
Hs_beta-arrestins in GPCR Desensitization (15) D4-GDI Signaling Pathway (22)	0	0.95	-0.71	14.7

D4-GDI Signaling Pathway (22) Role of arrestins in the activation and targeting of MAP kinases (22)	0	0.94	-0.65	13.2

Eicosanoid Metabolism (25) Lysine_degradation (28)	0	0.94	-0.66	13.2

Regulation of hematopoiesis by cytokines (28) Monoamine_GPCRs (34)	0	0.92	-0.66	12.6

Aspirin Blocks Signaling Pathway Involved in Platelet Activation (35) D4-GDI Signaling Pathway (22)	0	0.90	-0.68	12.3

D4-GDI Signaling Pathway (22) RB Tumor Suppressor/Checkpoint Signaling in response to DNA damage (23)	0	0.79	-0.80	11.5

T Helper Cell Surface Molecules (16) TGF beta signaling pathway (34)	0	0.88	-0.64	11.4

D4-GDI Signaling Pathway (22) Trka Receptor Signaling Pathway (22)	0	0.84	-0.68	11.0

Aspirin Blocks Signaling Pathway Involved in Platelet Activation (35) Msp/Ron Receptor Signaling Pathway (14)	0	0.81	-0.72	10.8

Msp/Ron Receptor Signaling Pathway (14) Roles of arrestin-dependent Recruitment of Src Kinases in GPCR Signaling (28)	0	0.79	-0.72	10.5

# of OGs: number of overlapping genes in two gene sets, NM: normal muscle, DMD: Duchenne's muscular dystrophy, dZIS: difference of Z-transformed IS, (): number of genes in a gene set, Nonparametric p value < 8.0E-7.

Table 5 shows the major pathways in the DMD data analysis results (see Table S4 in additional file 1 for the comprehensive list). The *D4-GDI Signaling *pathway had the highest number of interacting pathways (*n *= 10). The *Monoamine_GPCRs *pathway was connected to three others, which was the second highest number of interacting pathways.

**Table 5:** Major pathways showing significant differential coexpression with the other pathways in DMD dataset.

Pathway Name	K	Sum(ZIS)
D4-GDI Signaling Pathway (30)	10	111.5
Monoamine_GPCRs (42)	3	30.6
Trka Receptor Signaling Pathway (13)	3	29.2
Aspirin Blocks Signaling Pathway Involved in Platelet Activation (29)	2	23.1
Msp/Ron Receptor Signaling Pathway (18)	2	21.4

*K*: number of pathways showing differential coexpression, Sum(ZIS): total sum of Z-transformed IS, (): number of genes in a gene set.

## Discussion

In the present study, we developed a method for identifying significant changes in expression similarity (or coexpression) of two gene sets under two different conditions.

An important feature of this method is the transformation of the similarity between multivariate expression matrices into a single IS (see Figure 1 and 2). By visually inspecting original expression matrices, it is hard to quantify the expression similarity between two gene sets or its conditional change. The IS makes the change scalable. The advantage of this multivariate approach becomes more apparent when compared with a single gene approach. Single gene differential coexpression analysis failed to identify significant gene pairs in either dataset. The dCoxS, however, successfully identified significant gene set pairs biologically relevant to the conditional changes.

The idea of measuring matrix similarity originated from Mantel statistics. The Mantel statistics measure the similarity of two matrices using the correlation coefficient of sample distances [18]. Instead of other well-known distance metrics, however, we used the quadratic Renyi relative entropy with multivariate kernel density estimation to compute sample distances. The Renyi relative entropy is calculated by subtracting each sample's Renyi entropy, which has a metric property [19]. Therefore, it is equivalent to the distances between samples. Because the entropy was estimated according to the multivariate density, it may elucidate the correlation structure between variables. Because gene sets are defined by biological knowledge, the member genes are likely to have an internal correlation structure. To model the correlation structure in a gene set, the Renyi relative entropy may be an appropriate distance metric.

The simulation study validated our assumptions. We tested whether the IS represents the similarity between two gene expression matrices. Because random values were generated with increasing SDs, it is likely that simulated matrices with higher SDs are more dissimilar to the original matrix. Therefore, ISs between the original and the simulated matrices should be lower at higher SDs. We found that the average IS decreased as the SD increased, and the differences of the mean ISs were significant (Table 1). This finding indicates that the IS may represent the expression similarity between two gene sets.

Second, we compared the IS and Mantel statistics using various distance metrics. All five distance metrics of the Mantel test use the sum of squared or absolute values of differences between the gene expression values of two sample vectors. Therefore, the same distances can be obtained even if the sample expression vectors have different combinations of up and down gene expressions. Under these conditions, the Renyi relative entropy determines different sample distances because the multivariate kernel density estimation was used for the entropy computation (see Additional file 2 for detailed examples). Thus, the similarity score from the Mantel statistics may be higher than the IS for the same data. As expected, all Mantel statistics were higher than the IS on average and the IS was the lowest score among the similarity measures in the simulation study (Table 1). Biologically, distances from a sample vector to two different sample vectors should be different. Therefore, we concluded that the IS a representative score for dissimilar gene expression matrices.

In this analysis, we applied both parametric and nonparametric methods for testing the significance of differential coexpression between two pathways. Because many p values were lower than the Bonferroni adjusted p values, we used strict threshold cutoffs for more reliable results. However, the correlation between the entropies may be different from the original assumptions of Fisher's Z statistics. Therefore, to check the selected pathway pairs, the results were reevaluated using a nonparametric permutation test. The nonparametric test also showed that the results were significant with 1.25 × 10^6 ^permutations in each dataset.

Lung cancer data analysis revealed many pathways related to the pathophysiology of lung cancer. For example, the *Cytokine Network *and *TNF/Stress Related Signaling *pathway pair showed the highest dZIS (Table 2). Many of the member genes of these pathways are known to be associated with squamous cell cancer of the lung [20,21]. The *Thrombin signaling and protease-activated receptors *pathway, which had the highest number of interacting pathways and sum of corresponding dZISs (Table 3), is known to be involved in the angiogenesis of lung cancer [22]. It is noteworthy that the *Cell Cycle: G1/S Check Point *and *Inhibition of Cellular Proliferation by Gleevec *pathway pair was detected (see Figure 2 and Table S1 in additional file 1). Although the Gleevec was originally developed for the treatment of chronic myelogenous leukemia, it has already been used for treating many kinds of solid tumors, including lung cancer [23]. This suggests a novel avenue for exploring the mechanism of Gleevec in solid tumors. We found a strong tendency that all pathway pairs had lower ISs in lung cancer samples than in normal samples (Table 2). This suggests perturbed normal molecular regulatory mechanisms in cancer.

DMD data analysis provided a new hypothesis about muscle cell degeneration mechanisms. The *D4-GDI Signaling *pathway showed the highest dZIS with the *Beta-arrestins in GPCR Desensitization *pathway (Table 4) and also had the highest number of interacting pathways (Table 5). From previous research, *D4-GDI *is known to be associated with cytoskeletal changes in apoptotic cells [24]. Therefore, a significant change of the IS with the other pathways implies that the *D4-GDI Signaling *pathway has an important role in propagating the abnormal genetic features of DMD. While all ISs in the lung cancer dataset showed positive signs in the normal lung and lung cancer samples, the ISs of 23 pairs in the DMD dataset showed the opposite signs of the normal muscle and DMD samples (see Table S3 in additional file 1). This suggests different degrees of perturbation of gene expression between lung cancer and DMD.

The lung cancer dataset showed a much larger number of significant pathway pairs than the DMD dataset. Massive genetic alterations in cancer, including mutation, insertion, deletion, and translocation, may result in severe perturbations of gene and pathway regulations as shown in previous studies [20,25]. DMD, on the contrary, has only a mutated dystrophin gene, and the pathology is largely confined to muscle tissue. The lower number of results in the DMD dataset compared with lung cancer may be explained by the limited change of gene expression of dystrophin-related genes. Previous studies also support this assumption [26,27]. Thus the gap in the number of significant results in the present study may reflect the genomic alteration of the datasets.

Pathways often share common genes. We used two approaches for such cases: assigning and not assigning the shared members to one of the pathways (see methods). When we assigned the shared members to one of the pathways, there remained a subset in the other pathway. Therefore, any biological interpretation of the pathway pairs should be done carefully, especially when the common genes occupy a large portion of the original pathway. This approach can be used to find a novel subset of a pathway that is differentially coexpressed with another pathway when two pathways have shared members. The discovery of the novel substructures of pathways will be investigated in further research.

## Conclusion

Here we proposed the dCoxS algorithm to determine gene set pairs showing significant differential coexpression under different conditions. The coexpression relationships between gene sets can be used to understand the biological mechanisms caused by conditional changes.

## Methods

### Simulation datasets

To validate whether the IS represents the similarity between two gene expression matrices, we generated dissimilar expression matrices using an original expression matrix. After selecting a pathway expression matrix from real datasets, we added random values to each element of the original expression matrix.

(1)

In the above equation, *SX*_*ij *_and *X*_*ij *_indicate the *j*-th gene expression values of the *i*-th sample in the simulated and original expression matrices, respectively. Random values were generated from the normal distributions (*μ *= 0) with different standard deviation (SD = *σ*) values. Consequently, as the SD increases, the similarity between the original and simulated matrices decreases. Six different SD values were applied (SD = 0.05, 0.1, 0.2, 0.3, 0.4, and 0.5) in this analysis; that is, one original matrix had six dissimilar matrices and corresponding ISs. This procedure was repeated for 700 pathway expression profiles from two real datasets (see below). ISs were calculated between the original and dissimilar matrices, and the means of the 700 ISs originating from the different SDs were compared.

In addition, using the veganR package, the Mantel statistics between the matrices were computed with five distance measures: Bray, Canberra, Euclidean, Gower and Manhattan. The IS and the five different Mantel statistics were then paralleled at each SD. For comparison, a paired t-test was used.

### Real datasets

We used two benchmark datasets, lung cancer and DMD microarray data. The lung cancer dataset, consisting of 17 normal lung and 21 squamous cell carcinoma samples, was downloaded from the Broad Institute [28]. The Affymetrix HU-95 Av2 platform was used for the lung cancer microarray experiment. In the DMD dataset (GSE1004), 11 normal and 12 DMD patient muscle samples used the same microarray platform [29]. The dataset was downloaded from the Gene Expression Omnibus (GEO) website . Robust Multichip Averaging (RMA) was applied to the datasets for microarray data normalization [30].

### Gene sets

Biological pathway information was used to define gene sets. We used the human biological pathways in the ArrayXPath knowledge base [31] and mapped the microarray probes onto the pathway nodes. In this analysis, we arbitrarily set the minimum size (i.e. the number of member nodes) of a valid gene set as 10 and found 350 pathways for the Affymetrix HU 95 Av2 platform.

### Overview of dCoxS

Figure 1 depicts the overall analysis flow of the dCoxS algorithm. Original expression matrices of two gene sets were transformed into sample pair Renyi relative entropy matrices for each condition. Consequently, two square matrices containing each sample pair's relative entropy were generated for each condition. Second, the IS, which is a correlation coefficient of the corresponding upper-diagonal elements of the two entropy matrices, was calculated for each condition. Finally, the statistical significance of the difference of the Fisher's Z-transformed ISs between the conditions was tested. For example, when we determined the IS of two pathway expression matrices with dimensions 25 by 20 and 15 by 20 (gene number by sample number) in a condition, we calculated 190 (= (20*19)/2) distances between samples for each pathway expression matrix. We then obtained the ISs by calculating correlation coefficients between the two distances.

### Coexpression of gene sets

To measure the degree of coexpression between gene sets, dCoxS used the variation of expression levels determined by calculating the Renyi relative entropy. The Renyi entropy is a generalized form of Shannon entropy [32]. It is given by equation (2), where *X *is a stochastic variable with a probability density function *fx*.

(2)

Because of its convenience of estimation from the data in a nonparametric manner, the quadratic Renyi entropy was used as a cluster evaluation function [19]. In the present study, we used the quadratic Renyi relative entropy to measure the distance between two samples (or hybridizations):

(3)

where  and  denote the probabilistic density of the different samples *i *and *j *in an estimated multivariate distribution from a gene set expression matrix. Since *α *= 2 and a sample was used, the log ratio of the density of the different samples approximated the quadratic Renyi relative entropy. Although log(*p*^*α*^) = *α*log(*p*), *α *is deleted because it has no effect on the calculation of the IS. The density was estimated using the Parzen window density estimation with the Gaussian kernel function. We used a multiplicative kernel for the density estimation, which can be expressed as:

(4)

where *d *is the number of variables, *n *is the sample size, and *K *denotes a univariate kernel function [33]. In this analysis, *n *is the number of samples in a condition, and *d *is the number of genes in a pathway. In equation (4), *X*_*ij *_is the expression value from the *i-*th observation of the *j*-th gene in a gene set expression matrix, and *x *is a vector containing the expression values of *d *genes in a sample. For bandwidth (*h*_*j*_) selection of each dimension, we used Scott's rule in equation (5) in which  is the estimated variance of the *j-*th variable [33].

(5)

For two gene sets under each condition, we obtained two relative entropy matrices of all sample pairs. In equation (6), as a measure of gene set coexpression under given experimental conditions, we defined the IS, which is the Pearson's correlation coefficient between the upper-diagonal elements of the relative entropy matrices of the two gene sets.

(6)

In the above equation, *RE*^*G*1 ^and *RE*^*G*2 ^are the matrices of the Renyi relative entropy of gene sets, *G*_1 _and *G*_2_, respectively. *RE*^*G *^is computed using the following equation.

(7)

### Differential coexpression of gene sets under different conditions

After computing IS, dCoxS used Fisher's Z-transformation of the IS in equation (8) to measure the differential coexpression of two gene sets under different conditions.

(8)

The p value of the difference in *Zf *values was calculated using the standard normal distribution.

(9)

*Zf*_1 _and *Zf*_2 _are the Fisher's Z-transformed values of the IS under two different conditions. *N*_1 _and *N*_2 _are the numbers of upper-diagonal elements of sample pair matrix, which is calculated by *n(n-1)/2 *(*n *= number of samples), for each condition.

In the differential gene set coexpression test, we obtained parametric p values and selected significant results according to the threshold determined from the p-value distribution. The selected pathway pairs were retested in a nonparametric manner. The nonparametric p value was determined by the number of cases where the difference of permuted ISs was larger than that of the original ISs. Permutation-based hypothesis testing was performed using two dimensions of permutation: gene-wise and sample-wise. Gene-wise permutation was conducted by randomly resampling an equal number of genes within each gene set. Shuffling sample class labels was performed for sample permutations.

(10)

In equation (10), *N *and *M *represent the number of gene and sample permutations, respectively. *dZf(IS*_*C*1_, *IS*_*C*2_*) *computes the absolute value of the difference of Z-transformed ISs from condition C1 and C2. *dZf*_*ij *_indicates the absolute value of the difference between *pIS' *and *pIS"*, which are ISs calculated from the *i*-th gene and *j*-th sample permutation. Gene- and sample-wise permutations generated a pair of random pathway expression matrices for each condition. After transforming raw expression matrices to the relative entropy matrices, the entropy matrices were permuted. Permuted ISs (*pIS' *and *pIS"*) were then computed with the permuted entropy matrices. This is the same nonparametric test method as that of the Mantel test [18]. *I(·) *is an indicator function. If the absolute value of the *dZf *of the permuted entropy matrices is larger than that of the original *dZf*, *I(·) *= 1. Otherwise, *I(·) *= 0. The number of permutations was 1.25 × 10^6^: 2,500 times for genes and 500 times for samples. We performed more permutations for genes because there were more probes than samples. All computations were performed using the R statistical package .

Pathway pairs often have common genes. In such cases, we used a different approach. For pathway pairs with no shared member, we directly applied the above method. For those pairs with shared members, we calculated three dZISs. One was computed by directly applying the above method without considering the overlapping genes (nonassigning method). The other two dZISs were obtained by assigning the shared genes to one pathway or the other (assigning method). If the common genes were assigned to one pathway, the genes were subtracted from the other pathway of the pathway pair that had common genes. The remaining genes of the other pathway were used in the computation of the IS. We chose the most significant dZIS as that of the pathway pair.

### Single gene pair-wise differential coexpression analysis

At first, all gene-gene correlation coefficients were calculated for each condition. Then, the conditional difference of the Fisher's Z-transformed correlation coefficients was tested for each gene pair (equations 11, 12).

(11)

(12)

In the above equations, *CC *indicates the correlation coefficient of single gene pair. *Zf*_1 _and *Zf*_2 _are the Fisher's Z-transformed correlation coefficients of conditions 1 and 2. *N*_1 _and *N*_2 _are the number of samples in conditions 1 and 2, respectively. From the normal distribution, p values for differential coexpression tests were obtained according to the difference between the *Z *values.

During calculation, three p values were obtained for each gene pair. The p values were those of correlation coefficients from condition 1 and condition 2, and from the difference between Fisher's Z-transformed correlation coefficients. Bonferroni multiple testing correction was applied to the p values, and gene pairs whose three p values were all lower than the Bonferroni adjusted p value were selected (adjusted p value = 6.274E-10 for 79,689,000 gene pairs).

## Availability and requirements

The R-code for the dCoxS algorithm is available on our supplementary website: .

## Authors' contributions

SBC designed the method and implemented the code. JK collected the gene sets and contributed to the discussion. JHK supervised this research and contributed to the discussion.

## Supplementary Material

Additional file 1 **This zip file contains four additional tables (Tables S1, S2, S3 and S4).** Click here for file

Additional file 2 **Equations of the five different distance metrics (Bray, Canberra, Euclidean, Gower and Manhattan) and additional explanations about their properties.** Click here for file
