# Supplementary material for: Identifying set-wise differential co-expression in gene expression microarray data
Source: BMC Bioinformatics. 2009 Apr 16;10:109. doi: 10.1186/1471-2105-10-109 (PMC2679020; doi:10.1186/1471-2105-10-109)
Supplement: Additional file 2 — Equations of the five different distance metrics (Bray, Canberra, Euclidean, Gower and Manhattan) and additional explanations about their properties. [file 1471-2105-10-109-S2.doc]

**Mantel statistics and 5 metrics (Bray, Canberra, Euclidean, Gower, and Manhattan)**

The 5 distance metrics can yield similar distances with different combination of up- and down- regulation of the genes in a gene set. For example, a gene expression sample vector (6,7,8), which consists of 3 genes, has the same distance with two different sample vectors (7,8,10) and (7,8,6) with the Euclidean distance. However, the last two vectors are mutually different in terms of gene expression. The third gene is up-regulated in the second vector and down-regulated in the third vector, compared with the other genes. This fact implies that co-variation of two distances from the same sample pair of distinct two gene sets tends to be more similar with the Mantel statistics than IS. The reason is as follows: Given that there is a sample-wise distance *d_A_* between sample 1 and 2 in a gene set A. If *d_B_* is a strongly co-varied distance of the same sample pair in a gene set B, the 5 distance metrics have higher probability to generate the distance *d_B_* because different sample vectors can yield equivalent distances, as shown in the above example.

Since the Renyi’s entropy does use the squared value of the difference between one sample and the remaining sample vectors in a gene set expression matrix, the distances between sample vector (6,7,8) and (7,8,10) and distance between (6,7,8) and (7,8,6) is different with the entropy. Therefore, the IS have higher probability to produce less co-varying distances than the other 5 metrics. Due to this property, similarity score from Mantel statistics can be higher than IS with the same data.

In above equations, *x_ij_* indicate gene expression value of *i*-th sample and *j-*th gene. Sum of squared or absolute difference of sample vectors is used for computation of the sample-to-sample distances. In Euclidean and Manhattan distance, sample distance will be the same if absolute differences of sample vectors are equivalent. In the other 3 distance metrics, absolute difference of sample vector is also used in the numerator. In denominator of the metrics, total sum of difference of two sample vectors and sum of difference between maximum and minimum value for each gene were used for Canberra, Bray and Gower distance, respectively. Different sample vectors can generate similar (co-varying) denominators if there is no significant change of the absolute difference because small changes can be ignored in total summation. Moreover, in Gower distance, gap between maximum and minimum gene expression value of a gene is robust to such change. Therefore, sample distances tend to be more equivalent with different sample vectors in Mantel statistics than IS. This would cause higher scores in the Mantel statistics with the same dissimilar matrix.
